# Supplementary material for: Progerin can induce DNA damage in the absence of global changes in replication or cell proliferation
Source: PLoS One. 2024 Dec 5;19(12):e0315084. doi: 10.1371/journal.pone.0315084 (PMC11620420; doi:10.1371/journal.pone.0315084)
Supplement: S1 Raw images — (PDF) [file pone.0315084.s001.pdf]

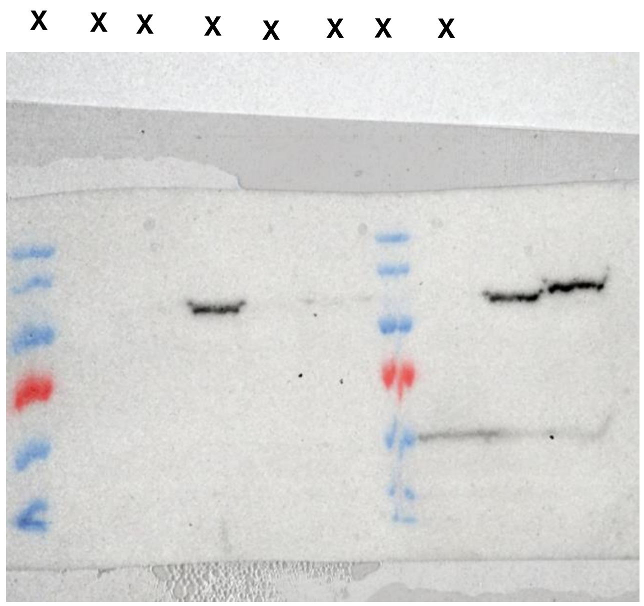

A cropped image of the two right-most lanes of this picture of a Western blot is shown in Panel B of Fig. 3. Samples were probed with an anti-GFP antibody. Thermo Scientific™ PageRuler™ Prestained Protein Ladder, 10 to 180 kDa, was used. Western blot images were analyzed using Typhoon FLA 7000 and ImageQuant LAS 4000 (GE Health).

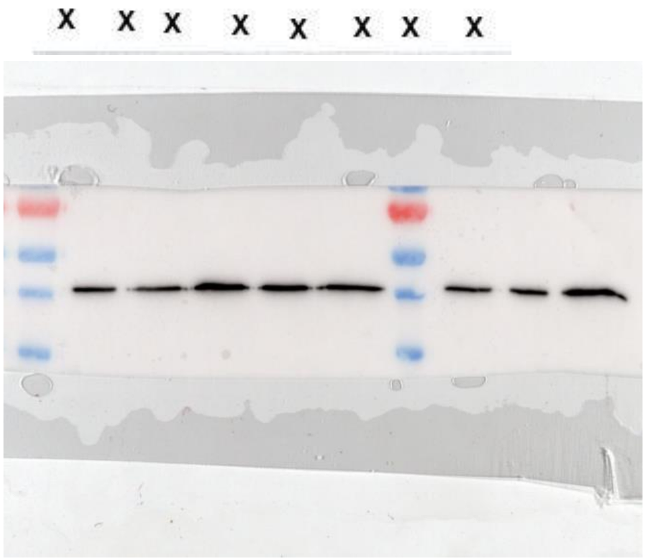

A cropped image of the two right-most lanes of this picture of a Western blot is also shown in Panel B of Fig. 3. This is a separate blot from the one probed with anti-GFP above and this blot was probed for beta actin. A separate blot was used because of the light spurious band that appears on the first blot at the same mw as beta actin. The Western blots were used simply to demonstrate that the two cell lines in the two right-most lanes express GFP-progerin and GFP-lamin A, respectively, at comparable levels.

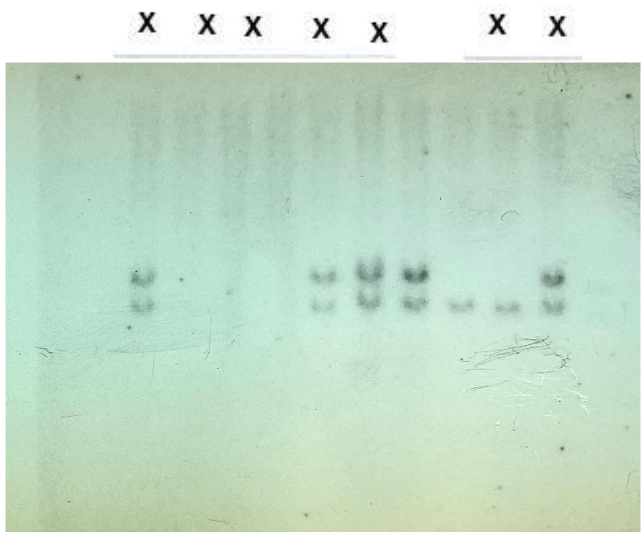

A cropped image of third and fourth lanes from the right of this picture of a Southern blot is shown in Fig. 7. These lanes display BamHI digestions of DNA from a crossover and a gene conversion event, respectively. The left-most visible lane in this picture, which is not shown in Fig. 7, represents a BamHI digestion of plasmid pLB4 and serves as DNA mw makers. The blot was developed manually using Amersham Hyperfilm MP and a picture was taken using an iPhone model XR.
